# Prototype and Chimera-Type Galectins in Placentas with Spontaneous and Recurrent Miscarriages

**DOI:** 10.3390/ijms17050644

**Published:** 2016-04-28

**Authors:** Laura Unverdorben, Thomas Haufe, Laura Santoso, Simone Hofmann, Udo Jeschke, Stefan Hutter

**Affiliations:** Ludwig Maximilians Universität München, Frauenklinik Campus Innenstadt, Maistrasse 11, 80337 München, Germany; laura.unverdorben@gmx.de (L.U.); thomas.haufe@googlemail.com (T.H.); laurasantoso@googlemail.com (L.S.); simone.hofmann@med.uni-muenchen.de (S.Ho.); stefan.hutter@med.uni-muenchen.de (S.Hu.)

**Keywords:** galectins, placenta, trophoblast, decidua, spontaneous and recurrent miscarriage

## Abstract

Galectins are galactose binding proteins and, in addition, factors for a wide range of pathologies in pregnancy. We have analyzed the expression of prototype (gal-1, -2, -7, -10) and chimera-type (gal-3) galectins in the placenta in cases of spontaneous abortions (SPA) and recurrent abortions (RA) in the first trimester. Fifteen placental samples from healthy pregnancies were used as a control group. Nine placentas were examined for spontaneous abortions, and 12 placentas for recurrent abortions. For differentiation and evaluation of different cell types of galectin-expression in the decidua, immunofluorescence was used. For all investigated prototype galectins (gal-1, -2, -7, -10) in SPA and RA placenta trophoblast cells the expression is significantly decreased. In the decidua/extravillous trophoblast only gal-2 expression was significantly lowered, which could be connected to its role in angiogenesis. In trophoblasts in first-trimester placentas and in cases of SPA and RA, prototype galectins are altered in the same way. We suspect prototype galectins have a similar function in placental tissue because of their common biochemical structure. Expression of galectin 3 as a chimera type galectin was not found to be significantly altered in abortive placentas.

## 1. Introduction

In pregnancy complex regulation of different immunotolerating systems is essential to avoid rejection of the fetus and subsequent miscarriage. Several causes for spontaneous abortion have been described and investigated. In most cases etiology is unknown, but several causes have been identified or proposed like placental anomalies, chromosomal [1], endocrine factors, lifestyle, and environmental factors [2,3,4]. Besides macroscopic factors like fibroma or malformations of the uterus [5,6], there are numerous features described on the molecular level. Interleukins and cytokines were described for interaction of cellular immune defense with T-cells. Elevated interleukin (IL)-15 expression in the extravillous trophoblast may lead to rejection in the process of nidation and vascularization [7]. Placenta-expressed cytokines like IL-2, tumor necrosis factor (TNF)-α, and Interferon (IFN)-γ are expected to disturb normal pregnancy development in the placenta, whereas T-helper2-cytokines (IL-4, IL-5, IL-6, and IL-10) may protect pregnancy in different ways and interactions [7,8]. Moreover other factors (e.g., thyroid hormone receptor expression) seem to be altered in spontaneous and recurrent miscarriages [9].

Therefore immunomodulating processes and interactions on the molecular basis seem to have various influences on the pathogenesis of abortion on different anatomic structures of the placenta, as it forms the bridge between the semi-allogenous fetus and the mother. Many of these pathologies are described for a third-trimester placenta; however, numerous pregnancies are failing long before, *i.e.*, shortly after implantation while the placenta is developing.

Some immunomodulating aspects concerning the placenta are attributed to the family of galectins. Galectins are mammalian β-galactoside-binding lectins that recognize galβ1-4GlcNAc sequences of cell surface oligosaccharides [10,11,12]. Recently, a growing number of galectins was described in various human tissues. The function of the galectins reaches from immunomodulation to regulation of metabolism [13,14]. Galectins are expressed by all immune cells, and they are upregulated in activated B and T cells, inflammatory macrophages and decidual natural killer (NK) cells [15,16,17]. Because galectins play a role in immunotolerance, they also influence pregnancy outcome [15,16,17] or gestational diseases like intra uterine growth restriction (IUGR) [18], preeclampsia [19,20,21], gestational diabetes mellitus [22], or spontaneous or recurrent abortion [12].

Consisting of a highly conserved amino acid sequence motif in the globular galectin-type Carbohydrate Recognition Domains (CRD), galectins have β-galactoside binding affinity [10]. Therefore galectins are classified into three types on the basis of their structural architecture of CRD: prototype, chimera, and tandem-repeat types [12,23]. As “prototype” galectins, gal-1, -2, -5, -7, -10, -11, -13, -14, and -15 are known. Only galectin-3 is described as a chimera-type galectin. Gal-4, -6, -8, -9, and -12 are tandem-repeat galectins [24,25].

In a first-trimester placenta gal-1, a proto-type galectin, was found to be expressed mostly in the cytotrophoblast, while the syncytiotrophoblast is not immunoreactive against gal-1. Gal-1 was one of the first galectins described and therefore was characterized as a prototype galectin. The structure of gal-1 was exemplary for other galectins, which are included in the group of prototype galectins [26,27]. Decreased expression levels of galectin-1 (gal-1) correlate with higher numbers of fetuses lost in mice [16]; additionally, low levels of gal-1 in early pregnancy are assumed to be predictive of preeclampsia [28].

Moreover, gal-1 and gal-3 influence the development of the trophoblast. Gal-3 may compensate for reduced trophoblast invasion in cases of preeclampsia [29,30]. Though our knowledge of the function of gal-1 and -3 at the feto-maternal interface appears to be extensive [12], knowledge of the other human prototype galectins (gal-2, -7, and -10) at the feto-maternal interface and in the context of pregnancy outcome is rather low. Although various studies investigated galectins at the feto-maternal interface [12], a systemic investigation of galectins in first-trimester placentas is still lacking. In cases of miscarriage or recurrent abortion, knowledge about expression patterns of galectins in first-trimester placentas is still lacking. An additional question would be whether there is a predominance of immune-stimulating or -inhibiting factors resulting from galectin expression.

The aim of this study is a systemic analysis of the expression of the human prototype galectins gal-1, -2, -7, -10 and of the chimera type gal-3 in first-trimester (7th–14th week of gestation) abortive placentas after SPA [31] and RA. Placentas obtained from legal termination of pregnancy served as the control group.

## 2. Results

### 2.1. Patient Characteristics

We have analyzed tissue from pregnancies with spontaneous abortions (*n* = 9), from recurrent spontaneous abortions (*n* = 12), and from induced abortions as a control group (*n* = 15) (ctrl). The samples of the control group were placentas from normal pregnancies with induced abortion. All women were healthy; for clinical data, see Table 1.

### 2.2. Galectin Expression in First-Trimester Placenta with Spontaneous and Recurrent Abortion

#### 2.2.1. Galectin-1 Expression in the Villous Trophoblast Is Significantly Lowered in Comparison to Healthy First Pregnancy Placentas

**Figure 1:**
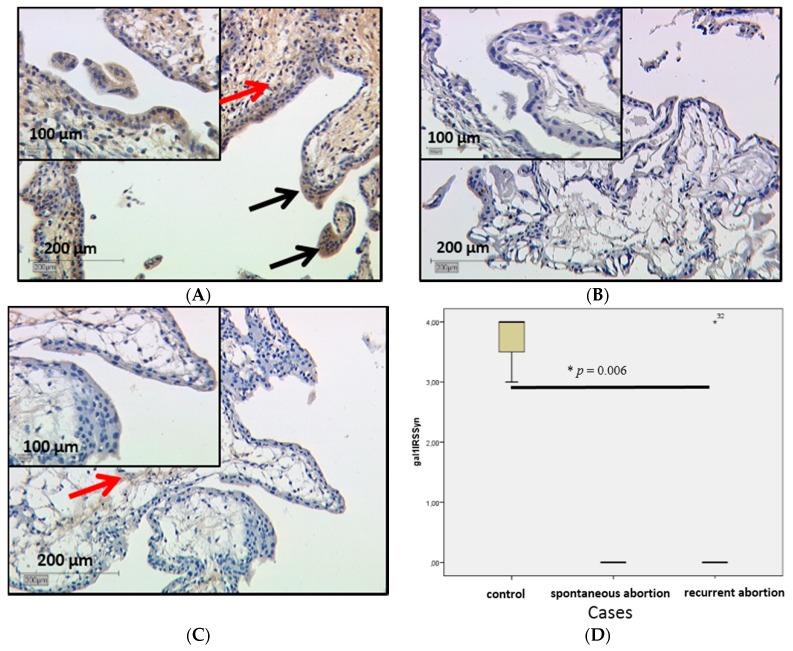
Immunohistochemical staining of gal-1 in the syncytiotrophoblast (marked by black arrows) and the villous stroma (red arrow) of first-trimester placentas: control placenta from induced termination of healthy pregnancy is shown in (**A**); IRS = 4. Spontaneous (**B**) and recurrent (**C**) abortion placentas are staining weaker in the syncytiotrophoblast as well as in the villous stroma (marked with a red arrow), Median IRS = 0 in both cases. The statistical results are shown in the boxplot (**D**). The boxes represent the range between the 25th and 75th percentiles with a horizontal line at the median. Bars represent the 5th and 95th percentiles. Asterisk marked with a number indicates values more than 3.0 box lengths from the 75th percentile.

Expression of gal-1 in the placenta was investigated by immunohistochemistry. In comparison to healthy first-trimester villous trophoblast (Figure 1A), gal-1 expression was significantly lowered in SPA and RA placentas with *p* = 0.006 in the trophoblast (Figure 1B,C respectively). In SPA and RA syncytiotrophoblasts, all cells were negative for gal-1 staining (International Remmele Score (IRS) = 0), while in control placentas IRS was 3.

Extravillous trophoblast and decidual staining showed no difference in RA and SPA placentas.

#### 2.2.2. In SPA and RA Placentas Expression of Galectin-2 Is Decreased in both Compartments

**Figure 2:**
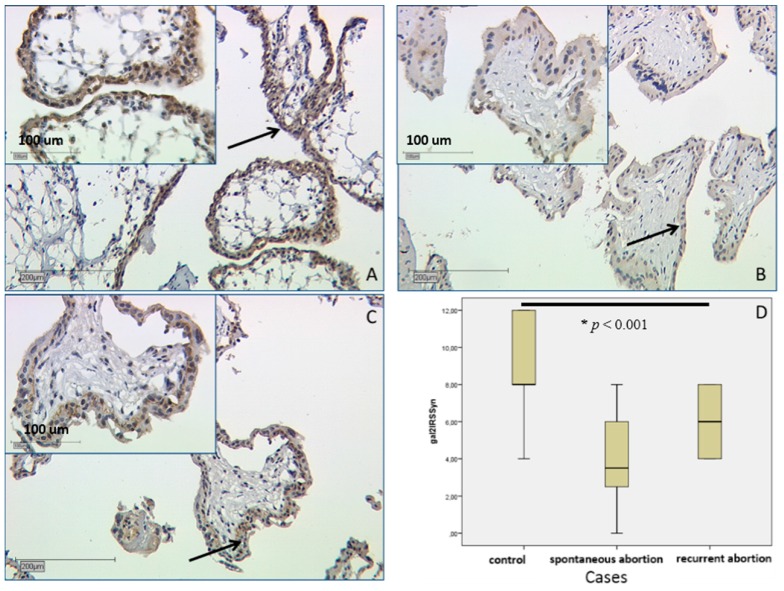
Galectin-2 expression in the syncytiotrophoblast (marked with a black arrow), as shown by immunohistochemical staining. Picture (**A**) shows strong staining in a control placenta (1st trimester) with median IRS = 8. Staining in the syncytiotrophoblast in case of spontaneous abortion is showed in (**B**) with IRS = 4 and in case of recurrent abortion in (**C**) with IRS = 6. The comparison of IRS results is shown in boxplot (**D**). The boxes represent the range between the 25th and 75th percentiles with a horizontal line at the median. Bars represent the 5th and 95th percentiles.

Evaluation of IRS scores showed a statistically significant lower level in SPA and RA placentas as well. This concerns both villous and extravillous trophoblasts. In the villous trophoblast of SPA placenta, staining was of medium intensity with IRS = 4 and in RA stronger with IRS = 6, compared to strong staining in the control group (IRS = 8) (Figure 2D). In a Kruskal–Wallis test this displayed significance with *p* < 0.001.

**Figure 3:**
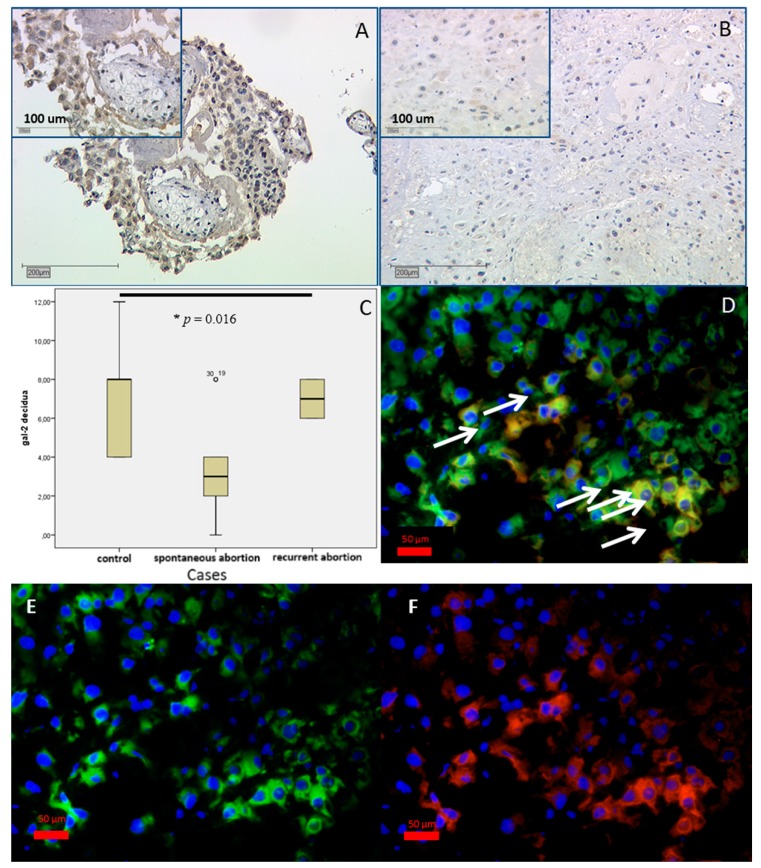
Galectin-2 expression was the only significant difference in the decidua of tested proto- and chimera-type galectins (*p* = 0.016). Immunohistochemical staining of gal-2 in extravillous trophoblast cells is shown in immunohistochemistry in the control (**A**) and spontaneous (**B**) abortion placenta with IRS = 3. In spontaneous and recurrent abortion, gal-2 IRS was significant lower than in control first-trimester placentas, as analyzed by box plot analyses (**C**); IRS = 8. Boxes represent the range between the 25th and 75th percentiles with a horizontal line at the median. Bars show the 5th and 95th percentiles. Values more than 1.5 box lengths are indicated by a circle. For differentiation of extravillous trophoblast and decidual stroma cells, immunofluorescence staining was performed. Triple filter excitation is shown in (**D**). HLA-G as trophoblast marker is appearing in green (**E**). Gal-2 appears in red (**F**); co-expression shows expression of gal-2 in extravillous trophoblast cells in yellow, as marked by white arrows.

Moreover, significant differences in the investigated galectin-2 staining were found in the decidua (*p* = 0.016). Gal-2 expression (Figure 3) decreased in spontaneous abortion, with a mean IRS of 3 (Figure 3A,D) in comparison to the strong staining in the control group (IRS = 8). Remarkably, in the RA group (Figure 3A), gal-2 staining was more intense, with mean IRS = 7 coming near the mean IRS of the control group (Figure 3A,C). As decidua consists of maternal decidual stroma cells and invading fetal extravillous trophoblasts, the different cell populations were differentiated with immunofluorescence. To identify these cells, extravillous trophoblast marker HLA-G was used. We found co-expression of gal-2 and HLA-G in the decidua (Figure 3B), so we can state that gal-2 expressing cells in the decidua appear to be extravillous trophoblast cells [32].

#### 2.2.3. Expression of Galectin-3 Is Not Altered in Abortive Placentas

**Figure 4:**
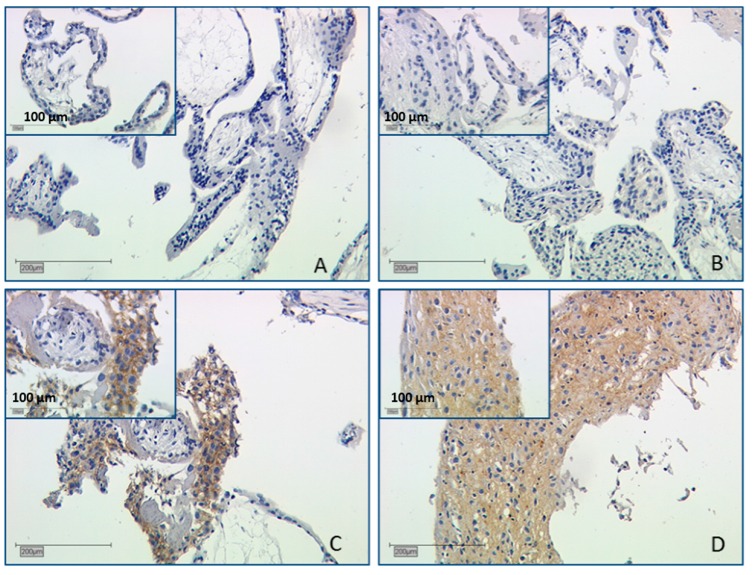
Immunohistochemical evaluation of staining intensity of gal-3 was not significant. (**A**) shows the staining in first-trimester control placentas; (**B**) shows gal-3 staining in the syncytiotrophoblast of spontaneous abortion placentas. Also, decidual staining was not significantly different between control (**C**) placentas (IRS = 3) and spontaneous (**D**; IRS = 4)) or recurrent decidual tissue.

Galectin-3 expression was also investigated by evaluating staining results. Here no significant alteration was identifiable in both SPA and RA collectives in villous and extravillous trophoblasts (Figure 4A,B). We could confirm the strict limitation of gal-3 staining to the trophoblast, while the syncytiotrophoblast was negative. In decidua, the control placenta’s (Figure 4C) staining was weak for gal-3 (mean IRS = 3). SPA and RA had moderate staining with mean IRS = 4 (Figure 4D), but with no significant difference to control placentas.

#### 2.2.4. Galectin-7 Expression Is Lowered in Villous Trophoblasts

**Figure 5:**
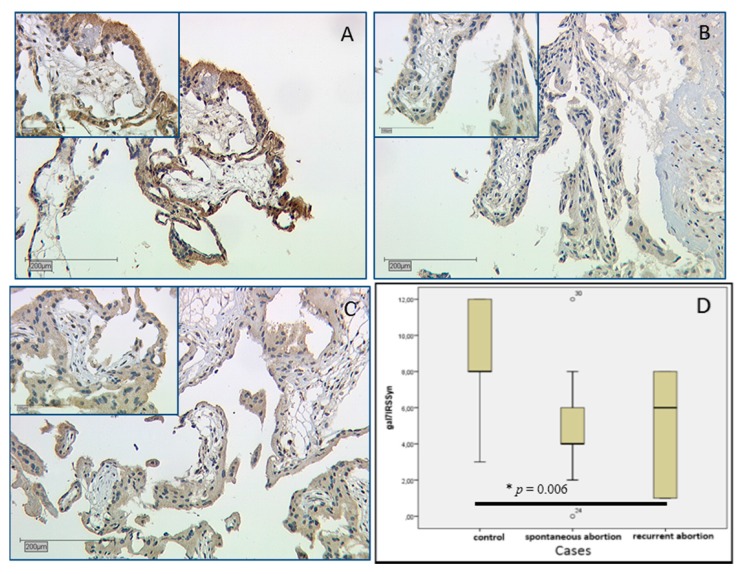
Immunohistochemical staining results of gal-7 correlating with its expression (all images have 10× magnification, inserts 25×): gal-7 staining in healthy control placentas is very strong with mean IRS of 8 (**A**). In spontaneous (**B**; IRS = 4) and recurrent (**C**; IRS = 6) abortion placentas gal-7 staining is significantly lower (*p* = 0.006). A summary is presented as a box plot in (**D**). The range between the 25th and 75th percentiles is represented by boxes with a horizontal line at the median. Bars show the 5th and 95th percentiles. Circles indicate values more than 1.5 box lengths.

Galectin-7 staining results showed significant differences in its expression in the villous trophoblast. In the control group strong staining with median IRS = 8 (Figure 5A) was detected, while anti-gal-7 displayed less staining in SPA placentas with median IRS = 4 (Figure 5B); in RA staining was slightly higher with IRS = 6 (Figure 5C). These differences are statistically significant (*p* = 0.006); all staining results are shown as boxplot (Figure 5D). In the extravillous trophoblast no statistical significant staining difference was found.

#### 2.2.5. In SPA and RA, Galectin-10 Is Lowered in the Villous Trophoblast

**Figure 6:**
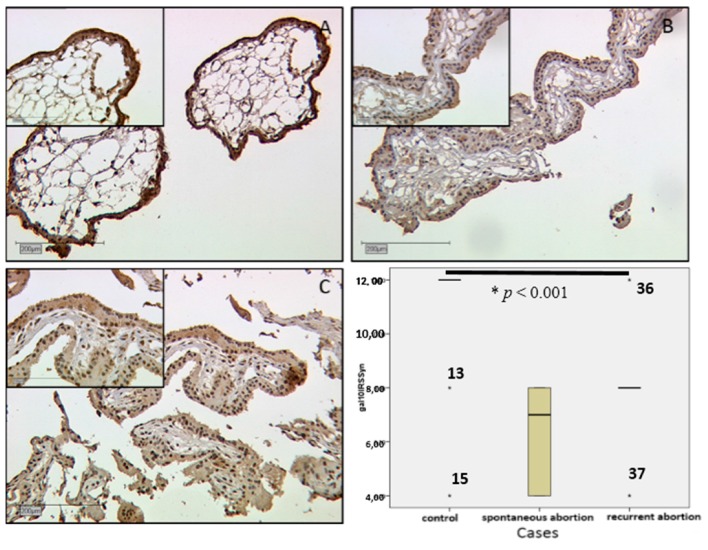
Galectin-10 expression is shown by immunohistochemical staining intensity in syncytiotrophoblast of first trimester placentas (all images at 10× magnification, inserts 25×). In control placentas of the first trimester, staining was very strong (**A**; IRS = 12). In cases of spontaneous abortion (**B**; IRS = 7) the expression was lower, as well as in cases of recurrent abortion (**C**; IRS = 8). IRS results are shown in box plot (**D**). Boxes represent the range between the 25th and 75th percentiles with a horizontal line at the median. Bars show the 5th and 95th percentiles. Asterisks marked with a number indicate values more than 3.0 box lengths from the 75th percentile.

Expression of gal-10 was investigated with staining results of immunohistochemistry. In villous trophoblasts, staining was very strong in the control group with median IRS = 12 (Figure 6A), while in the SPA group the median IRS was 7 (Figure 6B) and slightly stronger in the RA group with median IRS = 8 (Figure 6C). These differences were statistically significant (*p* < 0.001). Staining results are shown in the boxplot in Figure 6D.

### 2.3. Correlation Analysis

With Spearman’s rho test we searched for correlations between the different galectin expressions in IRS of syncytiotrophoblast cells.

There was no significant correlation between expression patterns of the galectins mentioned above.

## 3. Discussion

Galectins are described as a subfamily of animal lectins [10,23] with a common function of crosslinking galactose-containing structures found at cell surfaces and in the extracellular matrix of cells [33,34,35]. They are part of protein-to-protein interactions and influence modulation of cell growth, differentiation, and apoptosis [36]. Moreover, a role of galectins is assumed in neoplasia, but also in implantation in pregnancy [25,37]. With regard to the placenta, several galectins are described at different stages of gestation.

Due to the presence of two galectin-type CRDs in a single polypeptide or as a result of dimerization, most galectins have multiple sugar-binding sites [38,39,40]. As galectins are a large group of diverse proteins with different functions, we have investigated only prototype and chimera-type galectins according to their CRD architecture in first-trimester placentas [12,23]. These are galectins -1, -2, -7, -10 (prototype) and gal-3 (chimera-type).

### 3.1. Decreased Gal-1 Expression in the Trophoblast Is Correlated with Miscarriage

In placenta, gal-1 is found to bind to T-cells with immunoregulatory effect and therefore to interfere with the formation of syncytium [41,42,43]. In gestational trophoblastic diseases (GTD), immunoreactivity of both gal-1 and gal-3 was increased in the first trimester [44]. Gal-1 is known as a regulator of cell apoptosis, cell differentiation, and hormone synthesis [12]. It is described as a pivotal regulator of feto-maternal tolerance and might have potential therapeutic implications in threatened pregnancies, especially in trophoblast differentiation and in early pregnancy loss [16,45,46]. In first trimester and before implantation, serum-gal-1 levels are increased. Gal-1 is present in trophoblast cells of human blastocysts prior to implantation [28]. Moreover, expression of gal-1 in first-trimester placentas was located to the cytotrophoblast of the middle and distal cell columns differentiating toward fully invasive trophoblasts, while the syncytiotrophoblast was not immunoreactive against anti-gal-1-antibodies [28,47]. In our study we found negative staining results in the villous trophoblast of SPA and RA placentas. In contrast, staining was existent in control placentas. Diminished levels of circulating gal-1 correlate positively with subsequent miscarriage and make gal-1 a predictive marker for spontaneous abortion [28]. Additionally, we could prove that there is a lowered gal-1 expression in the placenta trophoblast. In decidua no gal-1 staining could be shown, similar to former findings of the invading cytotrophoblast only expressing low levels of gal-1 [30]. The villous trophoblast is known to secrete gal-1 as an immunosuppressive molecule, which was found to promote inhibition of T lymphocytes’ proliferation and of the adaptive immune response [48].

Gal-1 is described as having an anti-inflammatory effect and inducing tolerogenic dendritic cells at the feto-maternal interface, which can promote regulatory T-cells and IL-10-production to maintain and protect pregnancy [16,30,37]. Moreover, our study shows that a decreased expression in the villous trophoblast is correlated with adverse pregnancy outcome and miscarriage.

### 3.2. Gal-2 Is Expressed on Decreased Levels in Placentas after Spontaneous and Recurrent Abortion

Gal-2 is known to bind on beta1-integrins on T-cells, which can result in apoptosis of activated T-cells or show cell-specific responses on neutrophils [49,50,51,52]. In placental tissue its expression was previously described in induced abortion placentas on a moderate to strong level both in the villous trophoblast and extravillous trophoblast. The villous trophoblast showed strong staining at the brush border to the intervillous space. Thus far gal-2 expression was found to have decreased in third-trimester extravillous trophoblast (EVT) cells in cases of preeclampsia on the protein and mRNA level [21]. In our study, gal-2 expression was significantly lower in the villous and extravillous trophoblast of SPA and RA placentas.

As gal-2 is also described as an immunoregulative galectin and could play a role in angiogenesis, it is likely that a lack of gal-2 in the placenta (villous and extravillous trophoblast at the feto-maternal interface) may contribute to SPA and RA [21]. One explanatory idea concerning this aspect would be an overly active fetal immune response to maternal tissue or simply a reaction to the failed implantation.

### 3.3. Gal-3 as Chimera-Type Galectin Shows No Significant Difference of Expression in Spontaneous and Recurrent Abortions

Gal-3 is formed of two carbohydrate recognition domains and is currently the only known chimera-type galectin [12]. Lack of gal-3 in cells results in poor interaction with extracellular matrices, especially in epithelial tissue [12]. Furthermore, gal-3 is required for implantation in the endometrium of mice [53]. Gal-3 is localized in the villous cytotrophoblast and extravillous trophoblast [28,47]. Additionally, we have found weak gal-3 expression in healthy human first trimester placentas (7th–14th week). Staining appeared in both decidua and villous trophoblast, but with no significant difference in SPA and RA placentas. An inverse correlation between high gal-3 expression in the human placenta and trophoblast invasiveness has been further described for the course of gestation. Reduced trophoblast invasion in pathologic pregnancies complicated with preeclampsia and HELLP syndrome (H = hemolysis, EL =elevated liver enzymes, LP = low platelet count) may lead to compensatory elevated expression-levels of gal-3 in EVT [30,44]. However, in our study we cannot support these states for spontaneous and recurrent abortion in both trophoblast and extravillous trophoblast. It has to be remarked that spontaneous and recurrent abortions have different pathology and pathogenesis mechanisms.

Interestingly, all tested prototype galectins in this study are lowered in SPA and RA placentas in contrast to gal-3 as chimera-type galectin. Further investigations are needed to confirm this conclusion and to find out if this process can be reproduced (*i.e.*, on the mRNA level). Moreover we suspect that the structural differences between these galectin-types are interacting in the pathology of RA and SPA in diverse ways.

### 3.4. Gal-7 Expression Is Lowered in SPA and RA Placentas

Gal-7 was described in the endometrium of first-trimester placenta with immunohistochemistry, especially in syncytiotrophoblast in the placenta and at extravillous trophoblast in the cell column; furthermore, an adhesive role io gal-7 was described [54]. However, gal-7 also plays a role in the menstrual cycle and in endometrial epithelial wound repair *in vitro* [55].

In our study gal-7 was also found in the syncytiotrophoblast in first-trimester placentas after induced abortion, and with weaker staining in the decidua. In SPA and RA first-trimester placentas, the expression in the villous trophoblast/syncytiotrophoblast was significantly lower. An adhesive potential of gal-7 might have an influence on syncytium formation and immune tolerance during invasion of the trophoblast and decidualization [56]. Therefore, lower gal-7 expression in the villous trophoblast might lead to dysregulated immunotolerance of the invading trophoblast cells and may play a role in occurring abortion. Gal-7 levels are raised in serum probes of women and in the endometrium of women with a history of miscarriage [54]. Further results showed that serum galectin-7 was slightly elevated during the first trimester in women who developed preeclampsia later in pregnancy [57].

Gal-7 has been recently found to support tumor metastasis and progress in epithelial ovarian cancer. These effects are based on inducing apoptosis of lymphocytes and monocytes and a dose-dependent increase in the invasive behavior of cells *in vitro* [58]. Furthermore, gal-7 was shown to inhibit expression of interleukin-2 and interferon-γ mRNA in Jurkat cells and was proposed as a potential new immunosuppressive therapy against inflammatory skin diseases [59].

### 3.5. The Staining of Galectin-10 Is Strong, but Decreasing in SPA and RA Placentas

Gal-10, also known as eosinophil Charcot-Leyden crystal protein (CLC), has a similar structure compared to gal-13/PP-13 [60]. Gal-10 seems to be important for the functional properties of CD25(+)Treg cells and for differentiation of neutrophils [61,62]. So far it has been described in the colon with different diseases and in eosinophilic airway diseases [63,64,65]. In first trimester placenta a very strong staining in the syncytiotrophoblast and to a smaller extent in the decidua was found. In cases of SPA and RA, we found a significant decrease of gal-10 expression in the villous trophoblast. As for gal-10, an immunomodulatory role is assumed; a possible role of this galectin in the occurrence of miscarriage has to be further investigated, moreover, in interaction with described CD25(+)Treg cells, which also play a role in abortion [46,62].

## 4. Materials and Methods

### 4.1. Tissue Samples

A total of nine placentas were obtained from women treated after spontaneous abortion [31] and 12 after recurring abortion (RA) in the 1st department of Obstetrics and Gyaecology of LMU Munich. Placental tissue for the control group was obtained from 15 legal terminations of normal pregnancies because of socioeconomic reasons, reaching from the 7th to 14th gestational week (first trimester of gestation). Placental tissue was obtained after uterine curettage without hormonal pre-treatment. Demographic and clinical characteristics are presented in Table 1. Recurrent abortion (RA) is defined as three or more miscarriages before the end of the 16th week of gestation [7]. Women who suffered from SPA and RA were in the university medical care unit at their gynecologist during pregnancy, and operation was performed within 24 h after diagnosis.

The patients were screened for anatomic, chromosomal, and endocrine disorders. The inclusion criteria were the numbers of miscarriage and maternal age; exclusion criteria were thrombophilia, auto-immune diseases, hydatidiforme mole placentas, possible prostaglandin or progesterone treatment, or chromosomal aberrations.

Tissue was obtained in every case directly after delivery from three central parts of each placenta. Each block had to contain both decidua and trophoblast, proven by macroscopic inspection. For immunofluorescence, samples were immediately frozen in liquid nitrogen and stored at −80 °C for double immunofluorescence with cryosections. For immunohistochemistry, placental tissue was fixed immediately in 4% buffered formalin for 20–24 h and embedded in paraffin [30].

The study was approved by the ethics committee of the University of Munich (Project No. 337-06) at the 4th of January 2007 and informed consent to use the tissue was obtained from the patients in written form. For statistical workup, the samples and clinical information were anonymized.

### 4.2. Immunohistochemistry

For the staining procedure, the paraffin-embedded slides were dewaxed in xylol and washed in ethanol (100%). Inhibiting of endogen peroxidase of tissue samples was done in methanol with 3% H_2_O_2_ and afterwards slides were rehydrated in a descending series of alcohol. For gal-2, gal-7, and gal-10 staining, samples were heated in a pressure cooker using a sodium citrate buffer (pH 6.0) containing 0.1 M citric acid and 0.1 M sodium citrate in distilled water and after cooling were washed in distilled water and PBS. For gal-1 and gal-3-staining no heating was required and slides were incubated with horse serum (20 min, 22 °C; Vector Laboratories, Burlingame, CA, USA). A blocking solution (Reagent 1, Zytochem-Plus HRP-Polymer-Kit (mouse/rabbit), (Zytomed Systems, Berlin, Germany) was used for gal-2, gal-7, and gal-10 for incubation for 20 min.

The antibodies against galectin-2, -7, and -10 (Table 2) were incubated with the slides for 1 h at room temperature; gal-1 and gal-3 antibodies were incubated overnight at 4 °C.

Afterwards for gal-1 and gal-3 staining slides were incubated with the avidin–biotin–peroxidase complex (diluted in 10 mL PBS; Vector Laboratories, Biozol Diagnostica Eching, Germany) for 30 min. For the other galectins the different detection systems (Table 2) were used according to the manufacturer’s protocols.

Visualization of immunostaining was done with substrate and the chromagen 3,3′-diaminobenzidine (DAB; Dako, Glostrup, Denmark) for 30 s–2 min. Counterstaining of the tissue slides was performed with Mayer’s acid Hemalaun for 2 min with bluing in tab water. Afterwards, slides were dehydrated in an ascending series of alcohol. Following the treatment with xylol, slides were cover-slipped with Consul-Mount™ medium. (ThermoSherton, Pittsburgh, PA, USA).

**Figure 7:**
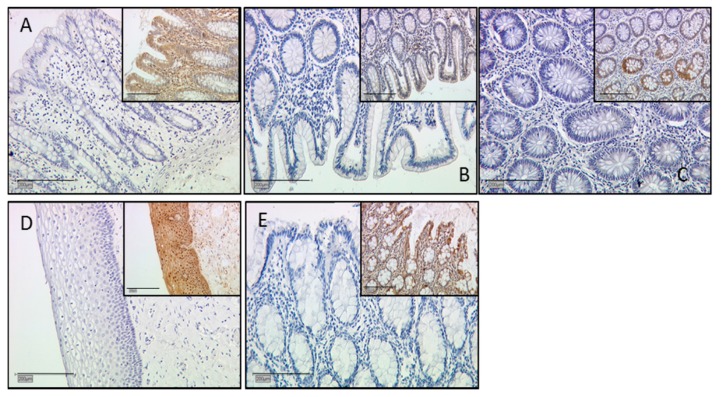

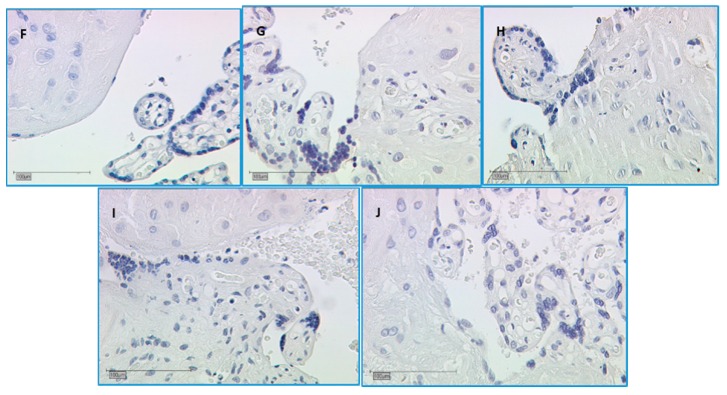
Negative control staining of galectin 1 in colon tissue (**A**) with a positive control insert of the same tissue, galectin 2 in colon tissue; magnification = 200 μm, inserts = 100 μm; (**B**) and positive control as insert, galectin 3 again in colon tissue; magnification = 200 μm, inserts = 100 μm; (**C**) and positive control insert, galectin 7 in vaginal tissue; magnification = 200 μm, inserts = μm; (**D**) and positive control insert in vaginal tissue and galectin 12 in sigmoid tissue; magnification = 200 μm, inserts = 100 μm; (**E**) and positive control insert, magnification = 200 μm; inserts = 100 μm. Negative control staining pictures for placental galectin expression are presented for gal-1 (**F**), gal-2 (**G**), gal-3 (**H**), gal-7 (**I**), and gal-10 (**J**), magnification = 100 μm.

For positive control tissue samples proven to be positive for the different galectin antibodies were used to perform immunohistochemical staining (Table 2). Positive cells showed a brownish color and the negative control, as well as unstained cells, appeared blue [66]. Negative controls were performed with the same control and placental tissue with subtype-specific control antibodies. For isotype control for gal-3 and gal-1 (mouse antibody), a mouse negative control antibody was used. Negative control staining is shown in Figure 7.

For analysis of the staining results a semi-quantitative score, the immunoreactive score (IRS) was used [67]. The IRS is the multiplication of the score of optical staining intensity (grades: 0 = none, 1 = weak, 2 = moderate and 3 = strong staining) and the score of percentage of positive stained cells (0 = no staining, 1 ≤ 10% of the cells, 2 = 11%–50% of the cells, 3 = 51%–80% of the cells and 4 ≥ 81% of the cells). This results in a score with a minimum of 0 and maximum of 12. Analysis was accomplished by two independent, well-trained staff members to rule out rater-dependent differences and inconsistency.

### 4.3. Double Immunofluorescence Staining

For the characterization of galectin-expressing cells in decidua, cryosections of the tissue samples of first-trimester abortion placentas were examined. The samples were fixed in acetone for 5 min. The antibodies used for the experiments are listed in Table 2 and Table 3. As a specific marker for extravillous trophoblast cells, HLA-G was used.

First, slides were blocked with Ultra V Block to avoid non-specific staining. Then, they were incubated with the gal-2 antibody and HLA-G (diluted 1:50 in Dako diluting medium, Dako) (Table 3 and Table 4) overnight at 4 °C. After washing with PBS in between each step, Cy2- and Cy3-labeled antibodies were applied as second antibodies. For gal-2, a Cy3-labeled antibody was used, appearing red; for HLA-G a Cy2-labeled antibody appearing green was applied and incubated for 30 min (Table 3). The slides were finally embedded in a mounting buffer containing 4′,6-diamino-2-phenylindole (DAPI) resulting in blue staining of the nucleus after washing and drying [68]. Finally, sections were examined with a Zeiss (Jena, Germany) Axiophot photomicroscope. With a digital camera system (Axiocam; Zeiss CF20DXC; KAPPA Messtechnik, Gleichen, Germany), digital images were obtained and saved on a computer.

### 4.4. Statistical Analysis

Statistical analysis was performed using SPSS 20 (PASW Statistic, SPSS Inc., IBM, Chicago, IL, USA) and Excel (Microsoft Windows 2013). The non-parametric Kruskal–Wallis rank-sum test was used to analyze the differences in galectin expression in abortion placentas among three or more groups. Spearman’s rho test was used for correlation analysis. *p*-values <0.05 were considered statistically significant.

## 5. Conclusions

We can state that in our study all investigated proto-type galectins (gal-1, -2, -7, -10) significantly decreased in SPA and RA placenta trophoblast cells. In the decidua/extravillous trophoblast no significant difference to the control tissue could be shown, except for gal-2, which could be based on its role in angiogenesis.

In the trophoblast of first-trimester placentas and in cases of SPA and RA, prototype galectins are altered in the same way. For now, we can suspect prototype galectins of having a similar function in placental tissue based on these findings.

Galectin-3, the only known chimera-type galectin, was not altered in cases of SPA and RA. Gal-3 is known as a predictor in cases of preeclampsia [30]. Further investigation and other methods are needed to explain the described expression patterns.

## Figures and Tables

**Table 1:** Demographic and clinical features of women part of the study. Values are given as mean ± SD; the range is given in parentheses. SPA = spontaneous miscarriage, RSA = recurrent abortion.

Characteristic	Normal Pregnancy *n* = 15	SPA *n* = 9	RSA *n* = 12	*p* Value (Kruskal–Wallis Test)
maternal age (years)	33.0 ± 6.7 (26–40)	31.5 ± 8.8 (19–43)	34.3 ± 4.6 (25–39)	0.813
gestational age (weeks)	9.1 ± 1.6 (7–14)	9.84 ± 1.4 (7–12)	8.7 ± 2.2 (7–11)	0.370
gravidity	3.2 ± 1.3 (1–6)	2.2 ± 2.6 (1–9)	2.9 ± 0.8 (2–4)	0.077
parity	1.6 ± 0.7 (0–3)	1.2 ± 2.6 (0–8)	0.7 ± 1.8 (0–2)	0.475

**Table 2:** Antibodies used in study for immunohistochemistry.

Antigene	Clone	Species and Isotype	Concentration/Dilution	Source of Ab	Detection System	Positive Control Tissue
Gal-1	K8508	goat	10 µg/mL 1:3000 in Power Block	R&D Systems, Minneapolis, MN, USA	Vectastain Elite Kit (Linaris, Dossenheim, Germany)	Colon, Mamma-Ca
Gal-2	H-45	rabbit	1:500 in Dako diluting medium	Santa Cruz, Dallas, TX, USA	Zytochem-Plus HRP-Polymer-Kit (Mouse/Rabbit) (Zytomed, Berlin Germany)	Colon
Gal-3	9C4	mouse	4.6 mg/mL 1:1000 in PBS	Novocastra, Wetzlar, Germany	Vectastain Elite Kit (Linaris)	Colon
Gal-7	H 60	rabbit	200 µg/mL 1:150 in Dako diluting medium	Santa Cruz	Zytochem-Plus HRP-Polymer-Kit (Mouse/Rabbit) (Zytomed)	Cervix
Gal-10	H-40	rabbit	200 µg/mL 1:100 in Dako diluting medium	Santa Cruz	Zytochem-Plus HRP-Polymer-Kit (Mouse/Rabbit) (Zytomed)	Placenta

**Table 3:** Supplement antibodies used in immunofluorescence.

Primary Antibody		Secondary Antibody
HLA-G, green	Mouse-IgG1 Clon MEM-6/9 (AbD Serotec, Puchheim, Germany) dilution 1:50 in Dako	Cy2-labeled Goat-Anti-Mouse IgG (Dianova Hamburg, Germany) diluted 1:100 in Dako → green
Gal 2, red	rabbit, (H-45) santa cruz	Cy3-labeled Goat-Anti-Rabbit IgG (Dianova) diluted 1:500 in Dako → red

**Table 4:** Kruskal–Wallis test: Significant differences in galectin staining in decidua and syncytiotrophoblast.

	Decidua	Villous Trophoblast			
*p*-value	gal-2	gal-1	gal-2	gal-7	gal-10
*p* (IRS)	0.016	0.006	0.001	0.006	0.001
